# Impact of Financial Incentives on Electronic Health Record–Driven Recruitment of Underrepresented Communities in Research: Randomized Controlled Trial

**DOI:** 10.2196/86110

**Published:** 2026-06-22

**Authors:** Christina Ping, Andrew A Davis, Waishun Gema, Foluso Ademuyiwa, Mark Fiala

**Affiliations:** 1 School of Medicine, Washington University in St. Louis, Saint Louis, MO, United States; 2 Division of Oncology, School of Medicine, Washington University in St. Louis, 660 S Euclid Ave, Saint Louis, MO, 63110, United States, 1 314-273-2783; 3 School of Public Health, Washington University in St. Louis, Saint Louis, MO, United States

**Keywords:** underrepresented racial or ethnic groups, electronic health records, research participation, patient portals, cancer

## Abstract

**Background:**

Progress in clinical trial research depends on effective recruitment to ensure adequate sample sizes and generalizability of results. Electronic health record (EHR)–based recruitment has been suggested as a potential method to address historical imbalances in trial populations, including underrepresentation of certain racial and ethnic groups. However, disparities in EHR portal uptake and use exist, particularly among these same underrepresented groups. Thus, we hypothesized that EHR-driven recruitment for research may continue to exacerbate these dynamics.

**Objective:**

In this study, we evaluated whether a financial incentive improved response rates to recruitment messages delivered through an EHR portal in an equitable fashion.

**Methods:**

A random sample of 1200 patients with diagnoses of breast, colorectal, prostate, or lung cancer and active EHR patient portal accounts received an automated recruitment message for a fictitious cross-sectional survey study, “The Social Aspects of Cancer Care,” via their EHR portal. Two strata of patients were sampled, those who identified as non-Hispanic White individuals (n=600, 50%) and those who identified as part of a historically underrepresented racial or ethnic group (n=600, 50%), and randomized to receive an offer of a US $20 monetary incentive (n=400, 33.3%) or no incentive (n=800, 66.7%). Patients were given 14 days to respond.

**Results:**

Overall, 39.3% (471/1200) of participants viewed the recruitment message, and 13.3% (159/1200) responded that they were interested. Response rates were higher (*P*<.001) among non-Hispanic White patients (108/600, 18%) than among African American or Black patients (32/456, 7%) and members of other racial or ethnic groups (18/144, 12.5%). Older patients (≥65 years) were less likely to view the recruitment message (244/720, 33.9% vs 230/480, 47.9%; *P*=.001) and respond (72/720, 10% vs 82/480, 17.1%; *P*=.002). Sex was not significantly associated with viewing the message or response outcomes (*P*=.45 and *P*=.19, respectively). No differences were observed in response rate for the incentive cohort compared to the cohort with no incentive (60/400, 15% vs 96/800, 12%; *P*=.21).

**Conclusions:**

Patients with cancer who identify as part of a historically underrepresented group were less likely to view and respond to recruitment messages for research studies through their EHR patient portal. Age was also found to significantly impact engagement, with older patients less likely to view and respond. Finally, a modest US $20 financial incentive was not found to significantly impact patient engagement.

## Introduction

Progress in clinical research relies on effective recruitment to ensure adequate sample sizes for statistical analyses and appropriate demographic makeups to support the generalizability of the final results [1]. Conventional recruitment strategies such as mail or phone contact or in-person recruitment with coordinators are costly and time intensive, which limits scalability [2-4]. Electronic health record (EHR)–based recruitment strategies have emerged as potential scalable tools to identify and contact eligible patients asynchronously [5].

Asynchronous digital techniques have been used for a variety of health-related campaigns [6]. Prior studies have used EHR portals to improve uptake for preventative screening [7], trigger vaccination reminders [8-10], and support research participant recruitment [11-13]. These studies have also demonstrated that using EHR portals to improve research engagement and participation is a cost-effective, easy-to-implement method of recruiting patients [11]. Some propose that EHR-based recruitment may be a solution to address underrepresentation of certain genders, races, or ethnic groups in clinical trials [12,13]. However, there is a concern that EHR-based recruitment strategies may exacerbate the well-documented disparities that exist among clinical research participants [14]. Data have shown that trial participants are more likely to be White individuals and older than the general population [15]. There is also evidence suggesting that disparities exist in patient virtual care and portal use by age, race, and socioeconomic status [16-19]. Missing from the literature are studies of EHR-based recruitment strategies that have compared the recruited patient sample to the overall population to evaluate whether EHR recruitment can actually address underrepresentation in trial participants. Furthermore, it is still unclear whether disparities in clinical trial engagement and participation based on race exist with EHR-based recruitment strategies. Some studies have reported that Black patients are equally likely to participate when asked and underrepresentation stems from lower access to research [20,21]. Another recent study found that Asian and Hispanic patients are less likely to view recruitment messages but Black and male patients are less likely to actually participate [22]. Thus, whether EHR-based recruitment is an aggravator of or solution to disparities in underrepresentation of these groups is still unknown.

Independent of EHR-based recruitment, financial incentives are also a strategy that has been shown to improve research engagement and participation [23]. Previous studies have found that financial incentives can improve response rates by a median of 12% [24], with the highest impact observed with direct monetary incentives [25]. However, their role in enhancing the representativeness of participant samples, particularly for EHR-driven studies, remains uncertain.

This study aimed to evaluate the effect of race and financial incentives on engagement with EHR portal recruitment messages among patients with and survivors of cancer. We hypothesized that (1) patients from historically underrepresented racial or ethnic groups that are enrolled in the EHR portal would be less likely to view and respond to recruitment messages and (2) a US $20 incentive would improve engagement, particularly among these patients.

## Methods

### Study Design and Participants

This randomized controlled study was conducted at the Siteman Cancer Center (SCC) under protocol 202402076 through the Hope Foundation for Cancer Research Pilot Grant Program. The SCC is a National Cancer Institute–designated Comprehensive Cancer Center that serves a geographically diverse catchment area, spanning across eastern Missouri and western Illinois. Approximately 22% of the SCC’s catchment identifies as being part of a historically underrepresented racial or ethnic group [26].

Eligible participants were defined as adults (≥18 years of age) with a diagnosis of breast, colorectal, lung, or prostate cancer documented in the EHR. These diagnoses were chosen because they are the 4 most common cancers in the United States. Only patients with active EHR portal accounts as of July 2024 and at least one clinic visit with either a medical or radiation oncologist within the prior 60 days were included.

We identified 5302 eligible patients, of whom 4399 (83%) identified as non-Hispanic White individuals and 903 (17%) identified as belonging to another racial or ethnic group (African American or Black, Hispanic, Asian, or other or unknown). From each race or ethnicity stratum, 600 patients were randomly selected. The purpose of oversampling patients who identified as being part of a historically underrepresented racial or ethnic group was to ensure that an effect size, if it existed, could be observed between the 2 groups. The primary objective of this study was to determine whether race impacted engagement with the recruitment message. With an α of .05 and 80% power, 800 patients were required to detect a difference of more than 5% in response rates. The secondary objective was to evaluate whether incentives changed these response rates. On the basis of a priori power calculations, we estimated that a sample size of 400 patients in the incentive cohort and 800 in the control cohort would allow us to determine whether there was more than a 10% increase in response rate between each incentive level compared to no incentives, with 80% power at a one-sided .05 α assuming an approximately 10% response rate in the control cohort. Thus, 66.7% (800/1200) of the recruited patients were randomized to receive no incentive, with the remaining 33.3% (400/1200) receiving a US $20 incentive alongside the recruitment message.

Randomization was completed via a random number generator and facilitated by author WG.

### Intervention

Patients received a standardized EHR portal message inviting them to participate in a fictitious cross-sectional survey–based noninterventional study, “The Social Aspects of Cancer Care.” Patients received an email notifying them of the invitation, and an alert was displayed on their landing page when they logged in to their EHR portal. Portal messages included details about the voluntary nature of participation, estimated survey duration, and incentive (if applicable). All patients who responded by clicking a button within the message stating “I’m interested” were subsequently debriefed about the sham nature of the study and given the US $20 incentive regardless of incentive group assignment. No additional data were collected from the patients who clicked on the link. Images of the email notification and portal alert can be found in Multimedia Appendix 1. Patients were given 14 days to respond to the invitation.

### Outcomes

The primary outcomes included message view rate, which was defined as the proportion of patients who opened the message in their patient portal, and message response rate, which was defined as the proportion of patients who responded to the message by clicking the “I’m interested” button embedded in the message. The secondary analyses examined demographic factors associated with engagement, as well as the potential impact of the incentive on the primary outcomes.

### Statistical Analysis

Descriptive and bivariate statistics were used to compare demographic characteristics by incentive and race groups. The primary end points were compared using Fisher exact tests. Multivariable logistic regression was also used to evaluate the independent associations among race, incentive, age, sex, and response outcomes. Adjusted odds ratios (aORs) and 95% CIs were reported. Statistical significance was defined as a *P* value of less than .05. Analyses were conducted using SAS Enterprise Guide (version 8.3; SAS Institute Inc).

### Ethical Considerations

This study was approved and supervised by the Washington University Human Research Protection Office. As participation was deemed to be of minimal risk and obtaining informed consent would have biased study results, a waiver of consent was granted.

## Results

### Participant Characteristics

A total of 1200 patients were randomized to receive a recruitment message through the EHR portal. The mean age was 65.7 (SD 12.3) years, and 54% (n=648) were female. Half (n=600, 50%) of the cohort identified as non-Hispanic White individuals, whereas 38% (n=456) identified as African American or Black individuals, and 12% (n=144) identified as being of other or unknown race or ethnicity. The most common diagnosis was breast cancer at 31% (n=372) of all patients. Prostate, lung, and colorectal cancer constituted 28% (n=336), 25% (n=300), and 16% (n=192) of patients, respectively. There were no statistically significant differences in age, sex, cancer type, or race distribution between incentive groups (*P*>.05 in all cases). Table 1 provides the detailed demographic characteristics of the study participants.

**Table 1.** Participant characteristics (N=1200).

	Overall cohort	No incentive (n=800)	US $20 incentive (n=400)	*P* value
Age (y), mean (SD)	65.7 (12.3)	65.7 (12.0)	65.7 (12.9)	
<65, n (%)	480 (40)	328 (41)	156 (39)	.99
≥65, n (%)	720 (60)	472 (59)	248 (62)	.45
Race, n (%)				.83
African American or Black	456 (38)	301 (38)	155 (39)	
Non-Hispanic White	600 (50)	400 (50)	200 (50)	
Other or unknown	144 (12)	99 (12)	45 (11)	
Sex, n (%)				.03
Male	552 (46)	392 (49)	168 (42)	
Female	648 (54)	416 (52)	232 (58)	
Cancer type, n (%)				.63
Breast	372 (31)	238 (31)	124 (31)	
Colorectal	192 (16)	136 (17)	60 (15)	
Lung	300 (25)	192 (24)	108 (27)	
Prostate	336 (28)	232 (29)	112 (28)	

### Participant Flow

**Figure 1.**
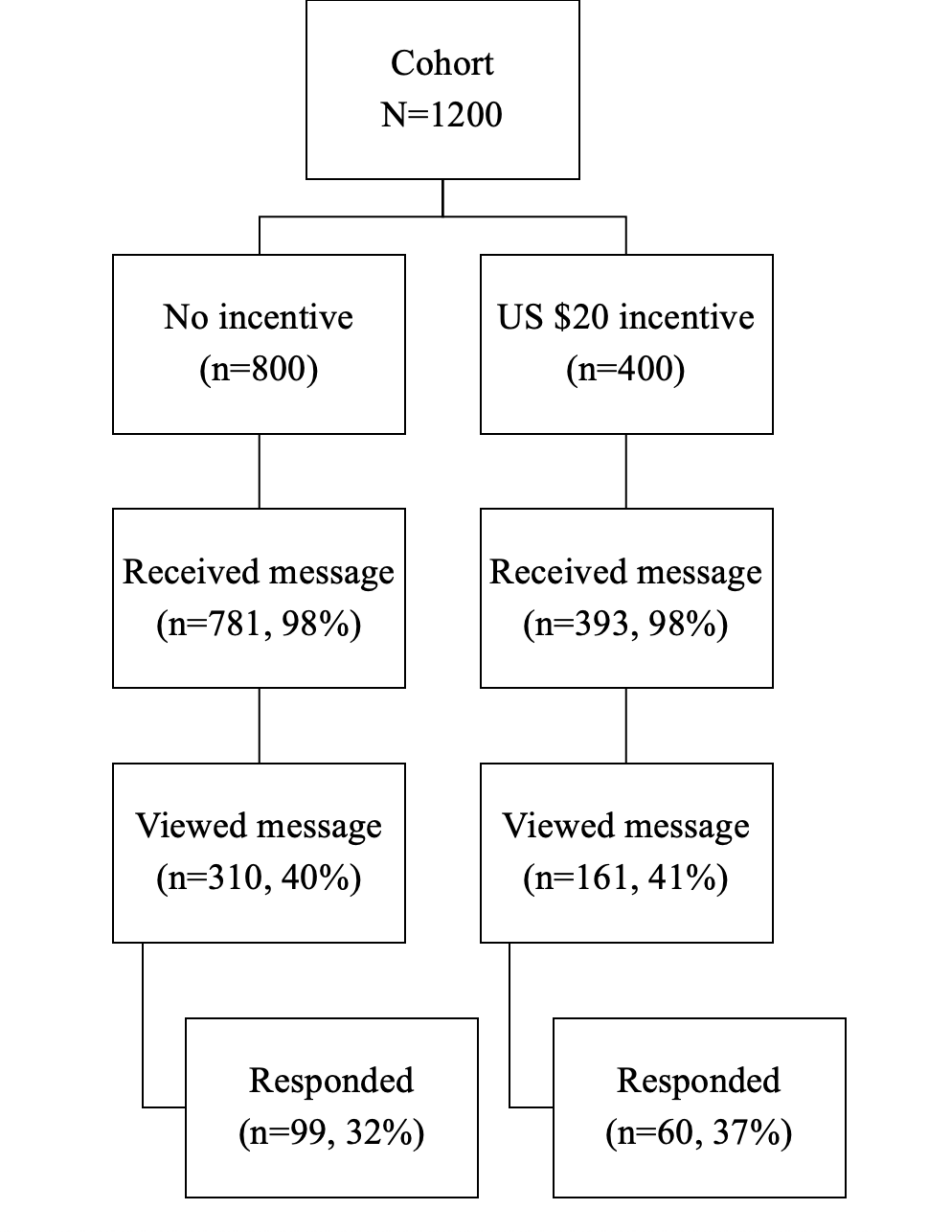
Participant flow.

Figure 1 shows the participant flow diagram. Message delivery was successful for 97.8% (1174/1200) of the participants. Unsuccessful deliveries were due to patient deaths (23/1200, 1.9%) or changes in account settings between participant identification and the message being sent (3/1200, 0.3%). Overall, 39.3% (471/1200) of the patients viewed the recruitment message, and 13.3% (159/1200) of the patients responded by clicking on the “I’m interested” button embedded in the message. Among those who viewed the message, the conditional response rate was 33.8% (159/471).

### Relationship Between Demographics and Response Rates

Table 2 details the results of our multivariable analyses on the effects of sex, race, and age on the primary outcome measures. Race was strongly associated with response to the recruitment message. Response rates were significantly higher (*P*<.001) among non-Hispanic White patients (108/600, 18%) than among non-White participants (60/600, 10%). Among non-White participants, African American or Black participants (32/456, 7%) had lower response rates than members of other racial or ethnic groups (18/144, 13%; *P*=.02). This finding was consistent for patients who did not receive an offer of an incentive (72/400, 18% in non-Hispanic Whites compared to 18/301, 6% in African American or Black and 10/99, 10% in other; *P*<.001) and for those who did (38/200, 19% in non-Hispanic Whites compared to 14/155, 9% in African American or Black and 9/45, 20% in other; *P*=.03). In multivariable logistic regression adjusting for age, sex, and incentive assignment, patients from historically excluded or underrepresented racial or ethnic groups had significantly lower odds of viewing the message (aOR 0.39, 95% CI 0.31‐0.50; *P*<.001) and responding (aOR 0.38, 95% CI 0.27‐0.55; *P*<.001). Among patients who opened the message, the conditional response rate remained significantly higher among non-Hispanic White patients than among patients from historically underrepresented racial or ethnic groups (110/275, 40% vs 51/232, 22%; *P*<.001).

**Table 2.** Multivariable analysis of factors impacting patient response.

	Message viewed (n=471 of 1200)		Responded (n=159 of 1200)		Responded (n=159 of the 471 who viewed the message)	
	aOR^a^ (95% CI)	*P* value	aOR (95% CI)	*P* value	aOR (95% CI)	*P* value
Incentive		.99		.21		.20
US $0	Reference		Reference		Reference	
US $20	1.07 (0.88-1.78)		1.25 (0.88-1.78)		1.31 (0.87-1.96)	
Race		<.001		<.001		.04
Non-Hispanic White	Reference		Reference		Reference	
Other	0.39 (0.31-0.50)		0.38 (0.27-0.55)		0.64 (0.42-0.97)	
Age (y)		.001		.002		.06
<65	Reference		Reference		Reference	
≥65	0.66 (0.52-0.85)		0.57 (0.40-0.81)		0.68 (0.45-1.02)	
Sex		.45		.19		.28
Female	Reference		Reference		Reference	
Male	0.91 (0.71-1.16)		0.79 (0.55-1.12)		0.80 (0.53-1.20)	

a aOR: adjusted odds ratio.

Older patients (≥65 years) were less likely to view the recruitment message (244/720, 34% vs 230/480, 48%; *P*=.001) and respond (72/720, 10% vs 82/480, 17%; *P*=.002). In multivariable models, older age remained associated with lower odds of message viewing (aOR 0.66, 95% CI 0.52‐0.85; *P*=.001). Sex was not significantly associated with viewing the message or with response outcomes.

### Effect of Incentives

**Figure 2.**
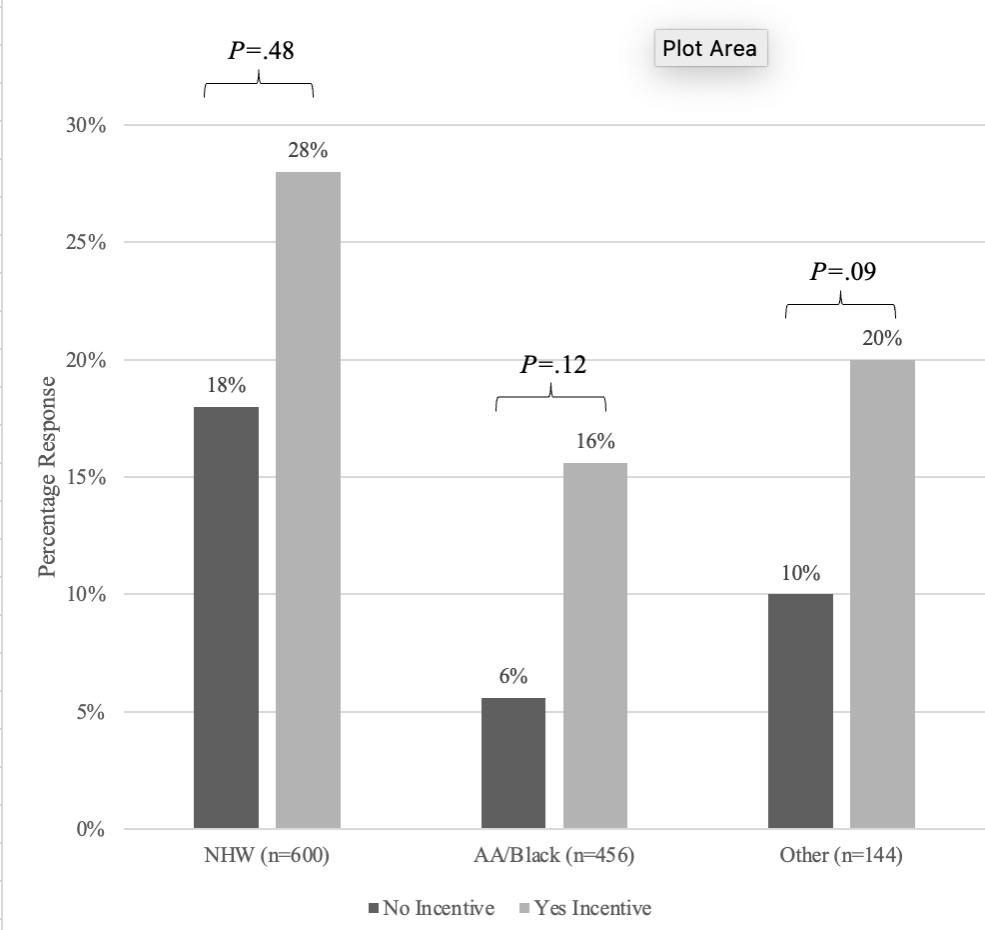
Response rate by race and incentive. The *P* values provided are one sided. AA: African American; NHW: non-Hispanic White.

Offering a US $20 incentive modestly increased the overall response rate (60/400, 15% vs 99/800, 12% in the no-incentive group), but this difference was not statistically significant (*P*=.21; Figure 2). Among patients who viewed the message, the response rate was similar across incentive groups (61/164, 37% vs 102/320, 32% in the no-incentive group; *P*=.20).

Exploratory subgroup analyses examined whether the incentive effect differed by race. Among patients from historically underrepresented or excluded racial or ethnic groups, the response rate increased from 7% (27/400) in the no-incentive group to 13% (26/200) in the incentive group. Among non-Hispanic White patients, the response rate was similar at 18% (72/400) in the no-incentive group and 19% (38/200) in the incentive group. However, there was no statistically significant interaction between race and incentive assignment (interaction *P*=.12).

## Discussion

### Principal Findings

We performed a randomized trial to examine the impact of EHR-based clinical trial recruitment with financial incentives on improving equitable representation in cancer research. We found that, among patients with and survivors of cancer who had active patient portal accounts, those who identified as part of historically underrepresented racial and/or ethnic groups were less likely to view and respond to recruitment messages for research studies, as we hypothesized. The results indicate that EHR-based recruitment may contribute to disparities in recruitment-stage engagement. In this study, we also found that the impact of the US $20 incentive on response rates was modest, suggesting that small, monetary incentives are insufficient to meaningfully improve recruitment. While there was a trend toward greater improvement in response rates after incentives among patients of other racial and ethnic backgrounds (increasing from 27/400, 7% to 26/200, 13% with the incentive), this did not reach significance in our sample (*P*=.12). Of note, this study was not specifically powered to detect the effect of incentives among different racial groups. However, this suggests that incentives may have differential effects depending on cultural context, perceived relevance of the study, or financial burden among underrepresented populations. Larger incentive amounts or more targeted financial incentives could be more effective in mitigating barriers to participation, such as incentives directly tied to transportation difficulties or food insecurity.

Furthermore, this study suggests that an EHR-based recruitment approach may be unsuccessful for older populations. Our findings demonstrated an impact of age on patient response even among patients with active user accounts. The mean age of the included participants was 65.7 (SD 12.3) years, and our results may have been skewed by this older patient base. It is possible that patient portal recruitment could have been more successful if conducted in a younger and likely more technologically adept population. This is in line with prior literature that has indicated that technological comfort and access decrease with age [27]. Recruitment for disease conditions that predominantly exist in older populations will likely need to use alternate recruitment strategies given this reduced comfort with technology.

Overall, our study demonstrates that asynchronous portal recruitment does not improve representation in trial engagement. Lack of diversity in clinical research limits the generalizability of trial results [14], and unfortunately, representation in research has only worsened over time in both industry-funded and academic center–sponsored trials [28]. The results observed in this trial may be due to the study not targeting specific barriers to engagement among patients from historically underrepresented racial or ethnic groups. Examples of such previously identified barriers include mistrust of health care institutions, structural factors that influence health literacy (eg, socioeconomic status and educational attainment), and logistical challenges to enrollment (eg, transportation) [29,30]. Other mechanisms of recruitment that specifically target these issues are likely needed to enhance overall representativeness of trial participants. For example, patient navigation programs targeting underresourced populations have been demonstrated to produce low trial refusal rates [31]. Similarly, in a survey of national clinical research sites, the practices found to be most impactful on diverse enrollment were travel reimbursement and services, translated documents and translator services, and adequate participant compensation [32]. While tailored recruitment strategies specific to each barrier may require more investment than a broader solution (such as EHR-based message recruiting), such investment may be necessary to truly achieve more equity among trial participants. This study suggests that racial disparities in trial enrollment may continue to worsen if EHR recruitment strategies are expanded [27].

This study has limitations. The single-center design may limit the generalizability of our findings when extrapolated to other populations as the demographic of patients in our catchment area is not necessarily representative of all US hospitals. Additionally, we specifically selected patients with active portal accounts. We have previously reported that patients who identify as being part of a historically underrepresented racial or ethnic group are less likely to have a patient portal account [18]. Furthermore, there was a racial and ethnic disparity observed in the initial eligible population (4399/5302, 83% non-White Hispanic patients vs 903/5302, 17% patients of other racial or ethnic categories) with active EHR portal accounts. There may be structural limitations or barriers (such as socioeconomic status or differential access to the internet) underlying this discrepancy that this study did not account for, which may have affected the engagement results observed in this study as well as limiting the potential generalizability of our results. It is possible that this study underestimated disparities in EHR-based recruitment.

While this study specifically focused on patients with a history of cancer, these findings may be generalizable beyond this patient population. Prior research has demonstrated that, while patients with cancer are as likely to enroll in clinical trials as the average American adult [33-35], they are less likely to have active patient portal accounts [36,37]. However, as our study only included patients with active portal accounts, the findings related to disparities in response are likely generalizable to other groups. This study contributes important evidence that EHR-based recruitment with or without modest financial incentives does not meaningfully reduce disparities in study enrollment, although our study suggests that the EHR could facilitate targeted recruitment of specific populations, including underrepresented communities.

Although our study focused on using patient portals for trial recruitment, these results may also suggest similar limitations when adapting patient portals for other use cases, such as behavioral interventions [38], telerehabilitation [39], or screening alerts [9]. Ultimately, we suspect that similar dynamics in message openings and patient response will persist in other clinically adjacent contexts. We recommend that future work investigate larger or targeted incentives, personalized outreach, and community-engaged recruitment and outreach approaches to enhance diversity and inclusion in both clinical research and therapy.

### Conclusions

In this randomized study, we found that patients with a history of cancer who identified as belonging to racial or ethnic minority groups were significantly less likely to view or respond to EHR portal–based research invitations. These findings reinforce long-standing concerns that digital tools alone, particularly those reliant on patient portals, may perpetuate or fail to mitigate inequities in clinical trial participation. More robust, culturally sensitive, and community-engaged approaches are needed to foster trust and facilitate engagement and participation among these populations. A small financial incentive only slightly increased overall response rates and did not meaningfully improve equity in research engagement in a survey-based study. Future research should focus on identifying and testing scalable, equity-focused recruitment models that balance efficiency with inclusivity, ensuring that all patients have a fair opportunity to contribute to and benefit from scientific progress.

## Supplementary material

10.2196/86110 Multimedia Appendix 1 Screenshot representative of the messages patients received inviting them to participate in the sham study. (A) Email notification. (B) Notification on MyChart homepage. (C) Research invitation message.

10.2196/86110 Checklist 1 CONSORT checklist.

## Abbreviations

aOR: adjusted odds ratio
EHR: electronic health record
SCC: Siteman Cancer Center

## References

[1] Tomiwa T, Wong E, Miller HN (2024). Leveraging digital tools to enhance diversity and inclusion in clinical trial recruitment. Front Public Health.

[2] Brøgger-Mikkelsen M, Ali Z, Zibert JR, Andersen AD, Thomsen SF (2020). Online patient recruitment in clinical trials: systematic review and meta-analysis. J Med Internet Res.

[3] Treweek S, Pitkethly M, Cook J (2018). Strategies to improve recruitment to randomised trials. Cochrane Database Syst Rev.

[4] Huynh L, Johns B, Liu SH, Vedula SS, Li T, Puhan MA (2014). Cost-effectiveness of health research study participant recruitment strategies: a systematic review. Clin Trials.

[5] Lai YS, Afseth JD (2019). A review of the impact of utilising electronic medical records for clinical research recruitment. Clin Trials.

[6] Frandsen M, Walters J, Ferguson SG (2014). Exploring the viability of using online social media advertising as a recruitment method for smoking cessation clinical trials. Nicotine Tob Res.

[7] Goshgarian G, Sorourdi C, May FP (2022). Effect of patient portal messaging before mailing fecal immunochemical test kit on colorectal cancer screening rates: a randomized clinical trial. JAMA Netw Open.

[8] Stockwell MS, Kharbanda EO, Martinez RA (2012). Text4Health: impact of text message reminder-recalls for pediatric and adolescent immunizations. Am J Public Health.

[9] Szilagyi PG, Albertin C, Casillas A (2020). Effect of patient portal reminders sent by a health care system on influenza vaccination rates: a randomized clinical trial. JAMA Intern Med.

[10] Lerner C, Albertin C, Casillas A (2021). Patient portal reminders for pediatric influenza vaccinations: a randomized clinical trial. Pediatrics.

[11] Samuels MH, Schuff R, Beninato P (2017). Effectiveness and cost of recruiting healthy volunteers for clinical research studies using an electronic patient portal: a randomized study. J Clin Transl Sci.

[12] Miller HN, Wong E, Byiringiro S (2025). CONNECT platform to increase diverse engagement and enrollment in cardiovascular clinical research: rationale, design, and lessons learned in early implementation. J Am Heart Assoc.

[13] Kannan V, Wilkinson KE, Varghese M (2019). Count me in: using a patient portal to minimize implicit bias in clinical research recruitment. J Am Med Inform Assoc.

[14] Unger JM, Vaidya R, Hershman DL, Minasian LM, Fleury ME (2019). Systematic review and meta-analysis of the magnitude of structural, clinical, and physician and patient barriers to cancer clinical trial participation. J Natl Cancer Inst.

[15] Brown EH Jr, Peiper NC, Furmanek S, McCants KC (2026). Integrating clinical trial data with electronic health records to improve diversity in research: findings from an urban hospital system. Trials.

[16] Graetz I, Gordon N, Fung V, Hamity C, Reed ME (2016). The digital divide and patient portals: internet access explained differences in patient portal use for secure messaging by age, race, and income. Med Care.

[17] Shah A, Fiala MA (2025). Disparities in access and utilization of electronic health record patient portals. J Gen Intern Med.

[18] Trammel AM, Sisk BA, Fiala MA (2025). Disparities in electronic health record patient portal activation and use among people with hematological malignancies. J Geriatr Oncol.

[19] Guetterman TC, Koptyra E, Ritchie O (2025). Equity in virtual care: a mixed methods study of perspectives from physicians. J Telemed Telecare.

[20] Unger JM, Hershman DL, Till C (2021). “When offered to participate”: a systematic review and meta-analysis of patient agreement to participate in cancer clinical trials. J Natl Cancer Inst.

[21] Walker DM, Swoboda CM, Shiu-Yee K, Tarver WL, Nolan TS, Joseph JJ (2023). Diversity of participation in clinical trials and influencing factors: findings from the Health Information National Trends Survey 2020. J Gen Intern Med.

[22] Navar AM, Esselink K, Lingvay I (2025). Response to electronic health record patient portal-based clinical study invitations. JAMA Netw Open.

[23] Singer E, Ye C (2013). The use and effects of incentives in surveys. Ann Am Acad Political Soc Sci.

[24] Anhang Price R, Quigley DD, Hargraves JL (2022). A systematic review of strategies to enhance response rates and representativeness of patient experience surveys. Med Care.

[25] Abdelazeem B, Hamdallah A, Rizk MA (2023). Does usage of monetary incentive impact the involvement in surveys? A systematic review and meta-analysis of 46 randomized controlled trials. PLoS One.

[26] PECaD dashboard: demographics. Siteman Cancer Center, Washington University School of Medicine.

[27] Gordon NP, Hornbrook MC (2016). Differences in access to and preferences for using patient portals and other eHealth technologies based on race, ethnicity, and age: a database and survey study of seniors in a large health plan. J Med Internet Res.

[28] Dunlop H, Fitzpatrick E, Kurti K (2022). Participation of patients from racial and ethnic minority groups in phase 1 early cancer drug development trials in the US, 2000-2018. JAMA Netw Open.

[29] Dawson S, Ostrovska B, Treweek S (2025). Sometimes different, often the same: guidance on how recruitment and retention in trials need to be tailored to enhance participation of ethnic minority groups: a qualitative interview study in England. J Clin Epidemiol.

[30] Thompson HS, Manning M, Mitchell J (2021). Factors associated with racial/ethnic group-based medical mistrust and perspectives on COVID-19 vaccine trial participation and vaccine uptake in the US. JAMA Netw Open.

[31] Ghebre RG, Jones LA, Wenzel JA, Martin MY, Durant RW, Ford JG (2014). State-of-the-science of patient navigation as a strategy for enhancing minority clinical trial accrual. Cancer.

[32] MacLennan DL, Plahovinsak JL, MacLennan RJ, Jones CT (2023). Clinical trial site perspectives and practices on study participant diversity and inclusion. Clin Pharmacol Ther.

[33] Unger JM, Shulman LN, Facktor MA, Nelson H, Fleury ME (2024). National estimates of the participation of patients with cancer in clinical research studies based on commission on cancer accreditation data. J Clin Oncol.

[34] Green AK, Tabatabai SM, Aghajanian C (2022). Clinical trial participation among older adult Medicare fee-for-service beneficiaries with cancer. JAMA Oncol.

[35] Williams CP, Senft Everson N, Shelburne N, Norton WE (2021). Demographic and health behavior factors associated with clinical trial invitation and participation in the United States. JAMA Netw Open.

[36] Griffin JM, Kroner BL, Wong SL (2024). Disparities in electronic health record portal access and use among patients with cancer. J Natl Cancer Inst.

[37] Maini I, Gilotra K, Sadigh G (2025). Patient factors associated with the use of online portal health information in the postpandemic era: cross-sectional analysis of a national survey. J Med Internet Res.

[38] Conroy SS, Harcum S, Keldsen L, Bever CT Jr (2022). Novel use of existing technology: a preliminary study of patient portal use for telerehabilitation. J Telemed Telecare.

[39] Steinhilber KM, Chabria RS, Clara A (2025). Shifting behavioral intervention research to virtual methods: challenges and solutions in practice, during and after the COVID-19 pandemic. J Telemed Telecare.

