# Supplementary material for: Impact of Financial Incentives on Electronic Health Record–Driven Recruitment of Underrepresented Communities in Research: Randomized Controlled Trial
Source: J Med Internet Res. 2026 Jun 22;28:e86110. doi: 10.2196/86110 (PMC13286524; doi:10.2196/86110)
Supplement: Multimedia Appendix 1 [file jmir-v28-e86110-s001.docx]

**
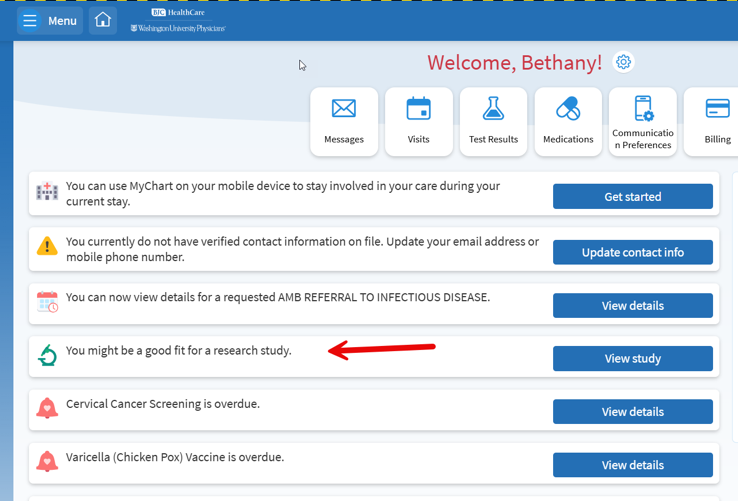

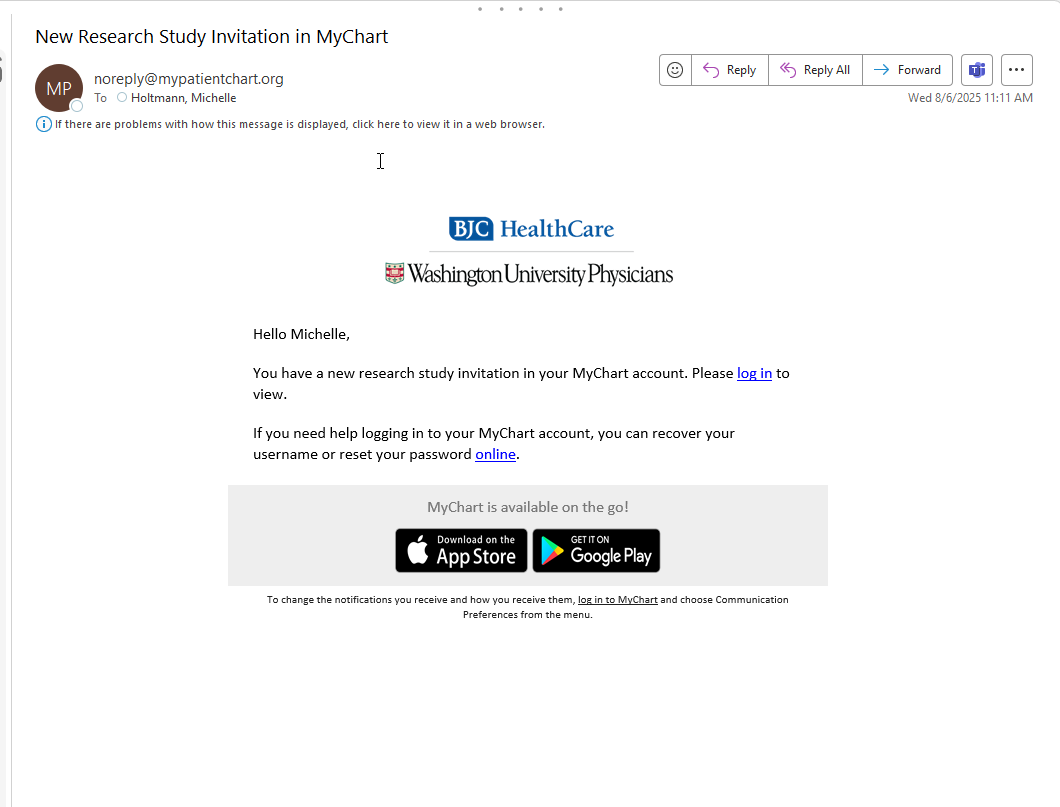
Supplemental Figure 1. Participant View of Sham Trial Invitation**

**A**

**B**

**
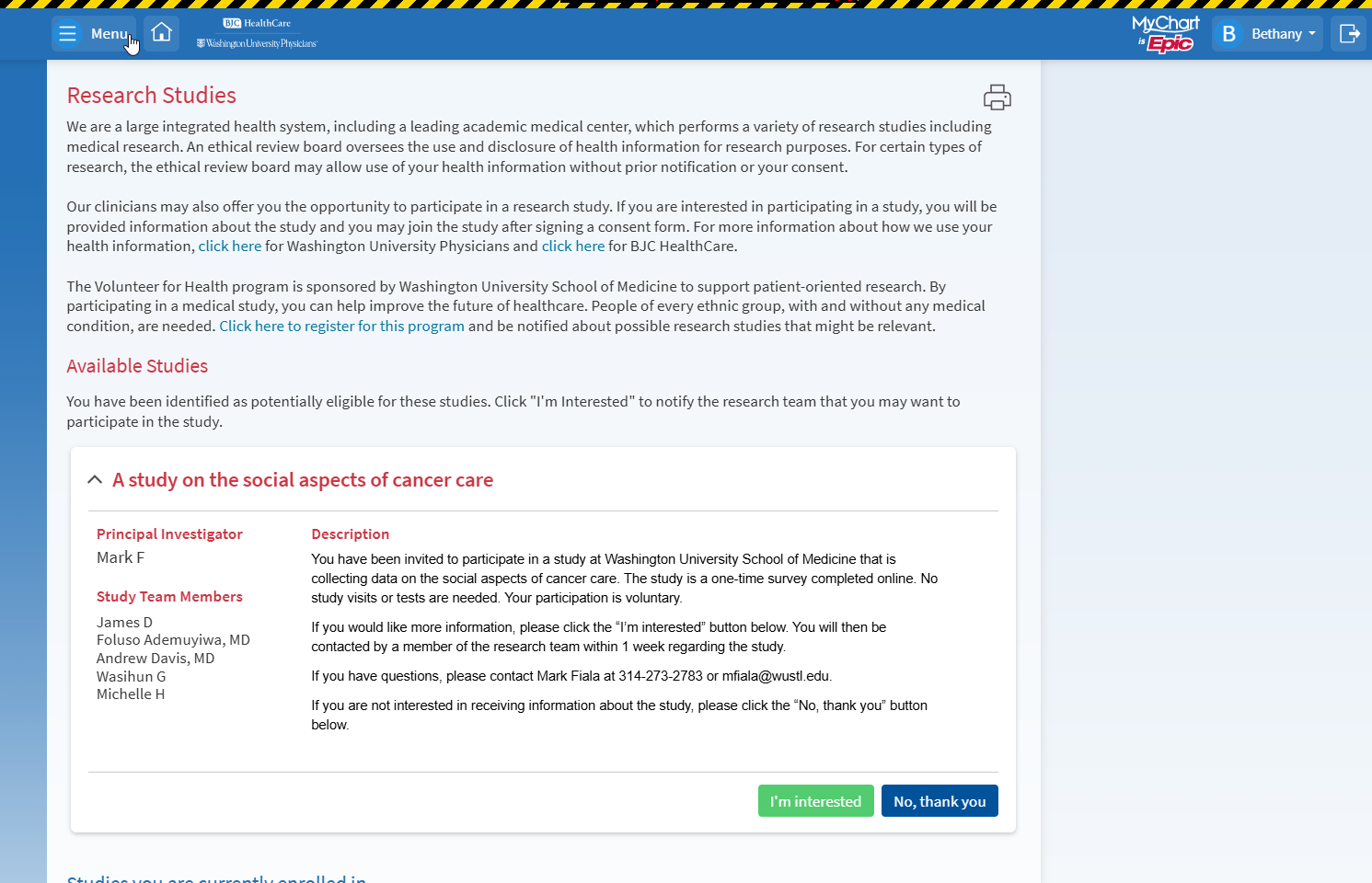
**

**C**

*Screenshot representative of the messages patients received inviting them to participate in the sham study. A: Email notification. B: Notification on MyChart homepage. C: Research invitation message.*
